# Isotropic Backward Waves Supported by a Spiral Array Metasurface

**DOI:** 10.1038/s41598-018-25469-7

**Published:** 2018-05-08

**Authors:** Ben Tremain, Ian R. Hooper, J. Roy Sambles, Alastair P. Hibbins

**Affiliations:** Department of Physics and Astronomy, University of Exeter, EX4 4QL Exeter, United Kingdom

## Abstract

A planar metallic metasurface formed of spiral elements is shown to support an isotropic backward wave over a narrow band of microwave frequencies. The magnetic field of this left-handed mode is mapped experimentally using a near-field scanning technique, allowing the anti-parallel group and phase velocities to be directly visualised. The corresponding dispersion relation and isofrequency contours are obtained through Fourier transformation of the field images.

## Introduction

The realisation of materials that exhibit negative refractive indices has been one of the most significant breakthroughs in electromagnetic metamaterial research in recent years. Such materials support modes whose phase and group velocities are in opposite directions (hence being described as ‘backward’ waves), can be described as effective homogenised media with simultaneously negative values of permittivity and permeability^1,2^, and exhibit negative refraction and perfect lensing effects^3^. Alternatively, these materials can be referred to as ‘left-handed’ due to the left-hand triad formed by the electric field *E*, magnetic field *H*, and wavevector *k*, of the waves. However, the backward wave description is preferred here to avoid confusion with chiral effects in metamaterials which can also be described in terms of handedness.

In 2000, Pendry and colleagues proposed a composite metamaterial designed to exhibit simultaneously negative effective permittivity and permeability^4^. An array of split-ring-resonators (SRRs) gave the material a resonant magnetic response (the material can be polarized by a magnetic field directed through the centre of each ring), whilst metallic wires produced a non-resonant electric plasma-like behaviour^5,6^. Within the frequency band of both negative permittivity and permeability, the material was shown to have a negative index of refraction^7^.

In addition to bulk waves, the surfaces of metamaterials support bound waves that are trapped at the interface between the metamaterial and surrounding medium. Such waves are also supported by single (or few) -layer metamaterials that are periodic only in two dimensions and often called metasurfaces. The propagating modes supported by the metasurface can be manipulated by varying the geometry of the subwavelength elements in much the same way that bulk modes are controlled in metamaterials, and we describe their phase velocity in terms of a *mode index*. The interest in metasurfaces is broadly split into two categories; the radiative properties (how incident planar waves interact with the structure) and the non-radiative properties (how trapped surface waves propagate across the surface). Frequency selective screens fall into the former category and can be used to filter out unwanted frequency bands for applications in the defence and telecommunications industries. Metasurface holography^8^ and actively tuneable beamsteering^9^ are other more recent areas of extensive research. In the non-radiative regime, lensing^10^ and beaming of bound surface waves has been studied, as well as various ‘cloaking’ devices^11^ which make use of the transformation optics approach to create a graded index surface. Metasurfaces have also been shown to support backward surface waves described as having a *negative* mode index^12^.

Modes guided by a metasurface can often be understood in terms of transmission line theory, where a backward wave medium is described as a resonant left-handed transmission line (LHTL)^13^ composed of series capacitors loaded with inductors in parallel. This has led to a number of studies on chains of split ring resonators acting as LHTLs^14–16^, where each element of the array can carry a circulating current that couples to its neighbours via a magnetic field. *Shamonina et al.*^17–19^ have produced several reports on these so-called magneto-inductive waves. Strong coupling is obtained when the rings are oriented to share a common axis, due to a large component of magnetic field directed through the ring centres. Weak coupling is observed when the rings are arranged in a planar configuration, however it is shown that in certain in-plane directions, the mode supported is a backward wave^18^. The dispersion curve of this mode shows that it has negative group velocity and positive phase velocity. Other studies have reported similar effects, all incorporating current-loop elements in a planar arrangement^20–24^.

Alternatively, such negative mode index behaviour can be explained in terms of an array of coupled magnetic and/or electric dipoles. Dipoles oriented longitudinally (transversely) to the direction of propagation result in a positive (negative) gradient in the dispersion curve^25^. It is the transverse case that is of interest to backward wave studies. The dispersion of the modes supported by an array of split-ring resonators has been theoretically analysed by simply describing the system as an array of out-of-plane magnetic dipoles and in-plane electric dipoles^26^. The analysis of magnetic dipoles in isolation demonstrated a negative gradient in their dispersion relation, however the realisation of such a system is difficult and limited to studies of high-index dielectric spheres^27^.

In this study, out-of-plane magnetic dipoles are created by using arrays of planar metallic spirals. In this geometry, current loops around the spiral produce a magnetic field through the centre that couples, albeit weakly, to neighbouring elements. By placing three-armed spirals in a hexagonal lattice, backward waves are supported in all in-plane directions to a high degree of isotropy. The structure can be viewed as a two-dimensional analogy of the negative index metamaterial^6^, and future studies using this metasurface design could demonstrate new opportunities for imaging and manipulation of surface waves, such as perfect lensing and negative refraction.

## Experiment

**Figure 1:**
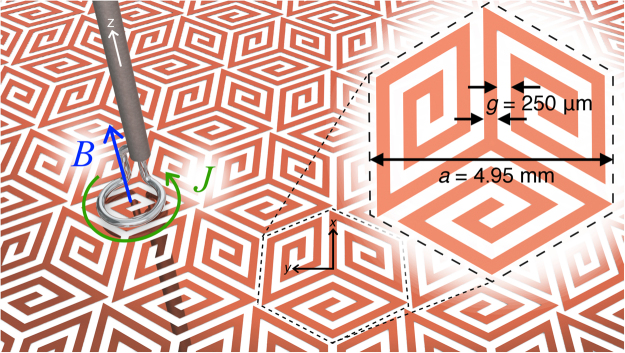
The spiral metasurface with unit cell geometry shown inset. The surface mode is coupled to the mode of a coaxial cable via a loop antenna positioned above the centre of a spiral arm. A second identical antenna scans the surface from the opposite side.

The proposed metasurface design (shown in Fig. 1) consists of a hexagonal array (pitch, *a* = 4.95 mm) of spiral-shaped copper elements, supported on a non-magnetic dielectric substrate (Mylar: ε = 2.8(1 + 0.03i)). Each spiral inclusion has three arms each with five turns. The arm width, *w*, and spacing between them, *g*, are both chosen to be 0.25 mm. The thicknesses of the metal and dielectric are 18 and 25 μm respectively. The eigenmodes and corresponding electromagnetic fields of the structure were simulated using COMSOL Multiphysics, where an infinite structure is modelled using the unit cell geometry (Fig. 1 inset) and periodic boundary conditions. A sample was subsequently fabricated using a ‘print and etch’^28^ method with overall size 400 × 280 mm.

Bound microwave surface waves were excited on the structure via coupling to the near-field of a loop antenna (shown in Fig. 1). The magnetic field, formed through the centre of the loop as a current is driven around it, couples to the out-of-plane component of the magnetic field of the surface mode when the antenna is placed adjacent to the surface (<1 mm). On the opposite surface a second loop antenna raster scans the surface and the transmission between the two antennas (mediated by the surface wave) is measured as a function of position. A Vector Network Analyser (VNA) and integral microwave generator sweeps through ‘continuous-wave’ frequencies in the range 5 to 15 GHz driving the source antenna. A Fourier transform of the resultant field maps for each frequency reveal the reciprocal space isofrequency contours from which the isotropy of propagation can be inferred (circular contours correspond to isotropic propagation). Since power must flow away from the source antenna, the group velocity of any waves must be in the positive radial direction, whilst the sign of the mode-index can be found by studying the propagation of the wave’s phase fronts. These will be away from the source for forward modes and towards the source for backward modes. For a given direction of propagation along the surface, a frequency-wavevector diagram of Fourier amplitude reveals the dispersion of the mode. A backward wave will manifest itself as a region of positive gradient (positive group velocity) for negative wavevectors (negative phase velocity), or vice-versa within the first Brillouin zone.

## Discussion

**Figure 2:**
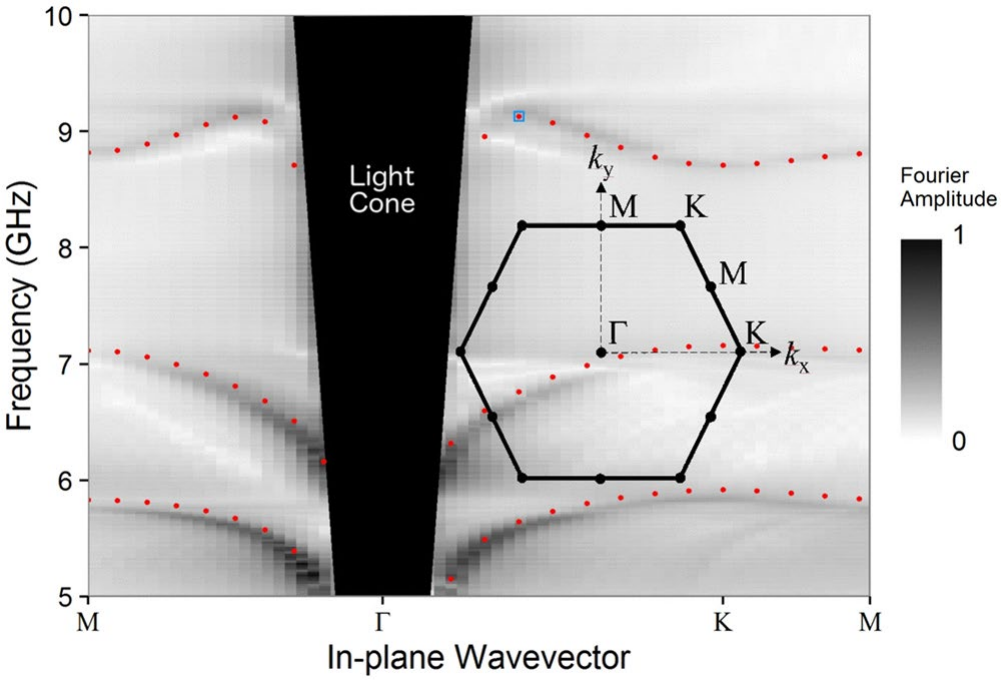
Fourier amplitude (arbitrary scale) of experimentally detected magnetic-field map across the spiral metasurface. This has been plotted as a function of in-plane wavevector and frequency in the first Brillouin zone (inset) to illustrate the dispersion of the supported modes. Modes within the light-line, corresponding to radiative modes, are beyond the scope of this work and have been omitted for clarity. The third-order mode has a backward wave solution as well as a positive wave solution.

The experimentally obtained dispersion curves are shown in Fig. 2, with results from numerical simulations overlaid. This dispersion covers the ‘irreducible Brillouin zone’, defined as a path through reciprocal space connecting the Γ, Κ, and Μ symmetry points. In both directions (ΓX and ΓM) there exists a band of frequencies for which the third-order mode has two solutions, one a forward wave, the other a backward wave.

**Figure 3:**
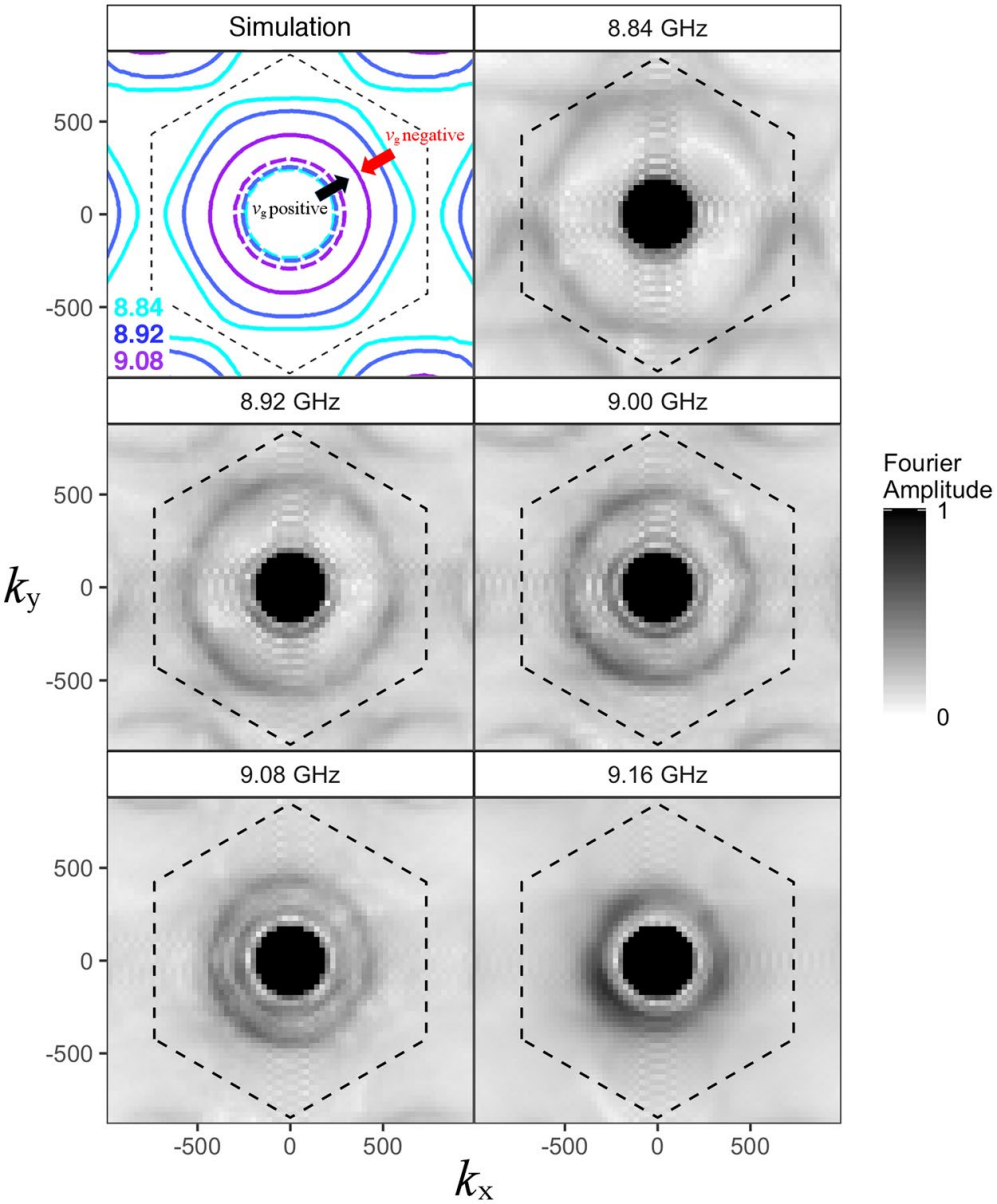
Top left: Simulated (FEM model) isofrequency contours in reciprocal space showing both the positive (forward wave, dashed lines) and negative (backward wave, solid lines) mode index surface waves. Other panels: Fourier amplitude (arbitrary scale) of experimental magnetic-field measured across the plane of the metasurface illustrating the k-dependence of the band as a function of frequency. The light circle is represented by the filled black circle.

This isotropy is more apparent in the isofrequency contours presented in Fig. 3, which show both forward and backward modes as approximately circular contours. The first panel shows the predictions from the FEM model of the contours as a function of increasing frequency; the behaviour of the positive and negative index modes is clearly evident. The experimental Fourier amplitude data is illustrated in the remaining panels, where the light cone is marked by the black circle. These results demonstrate a significant improvement in isotropy compared to previous studies of backward surface waves using Sievenpiper mushroom structures^12^.

**Figure 4:**
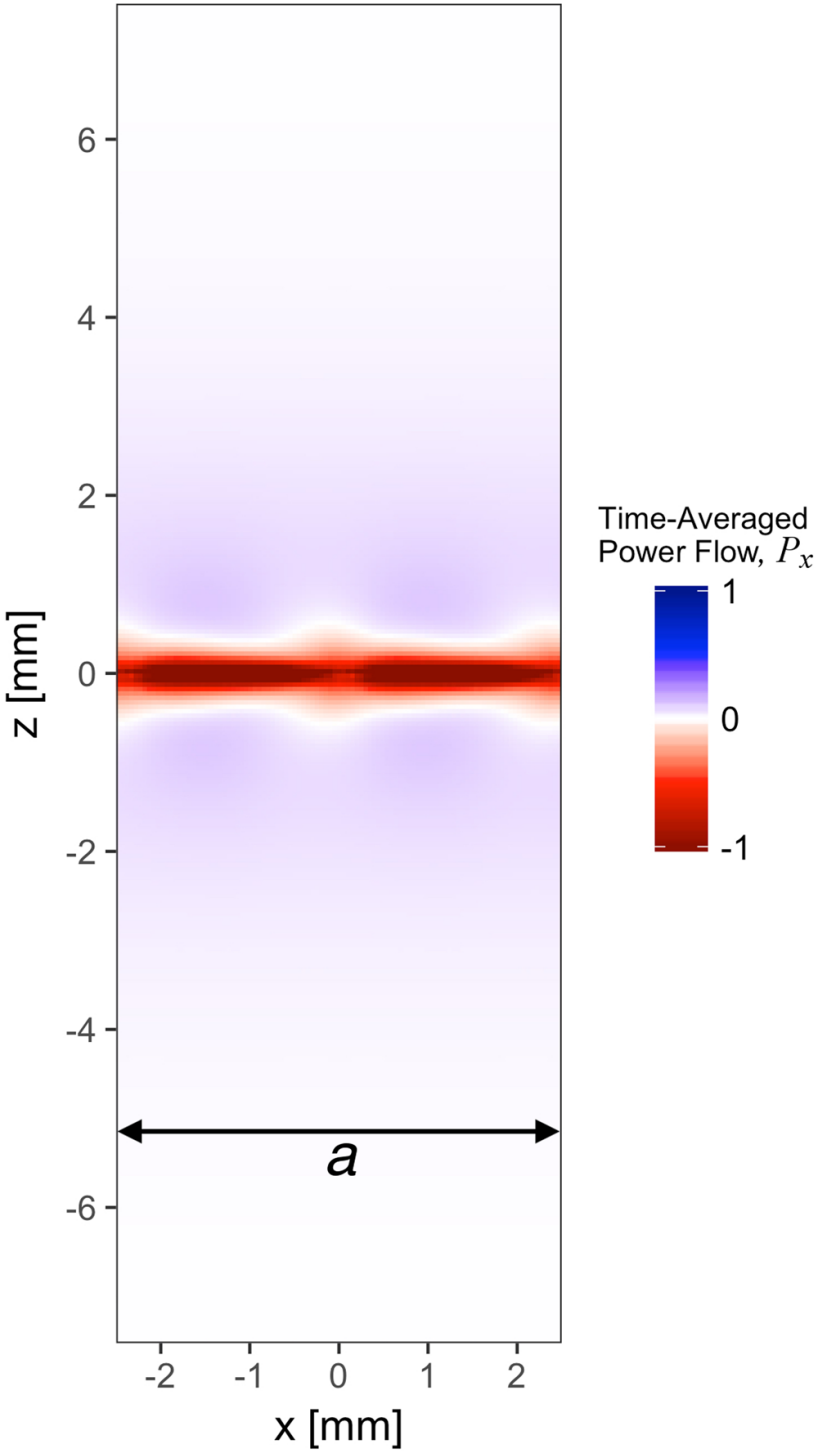
Simulated x-component of the time-averaged power flow (arbitrary scale), integrated in the *y* direction (out-of-plane), for the third eigenmode (*f* = 9.17 GHz) with in-plane wavevector of (*k*_x_, *k*_y_) = (350, 0) m^−1^. This corresponds to the blue square on Fig. 2, where the group velocity is zero. The sample lies in the *z* = 0 plane and the *x*-axis of the figure spans one unit cell.

In this report on mushroom structures, numerical modelling demonstrated that the time-averaged power flow was negative beneath the metallic patches, and positive above them. The direction of group velocity of the mode was given by the direction of net power flow through both of these competing regions. Similarly in the present case, despite the geometry being planar, two regions of power flow are observed. In Fig. 4 we plot the time-averaged power flow (integrated over all space in the y-direction) near the sample surface for the third Eigenmode at a value of |k| along ΓK at the turning point in its dispersion (blue square in Fig. 2). While the group velocity of the mode is zero, there exists an envelope of negative power flow in the immediate vicinity of the sample (located at z = 0), which is completely balanced by a surrounding region of positive power flow (|z| > 0.5 mm). The zero-group-velocity point occurs at the mode’s maximum frequency (Fig. 3; 9.16 GHz), and with decreasing frequency, we observe the evolution into backward- (high k) and forward- (low k) mode solutions. It is important to note that both solutions originate from the centre of the first Brillouin Zone |k| = 0, as is clear from Fig. 3, and are therefore not a consequence of diffractive coupling.

**Figure 5:**
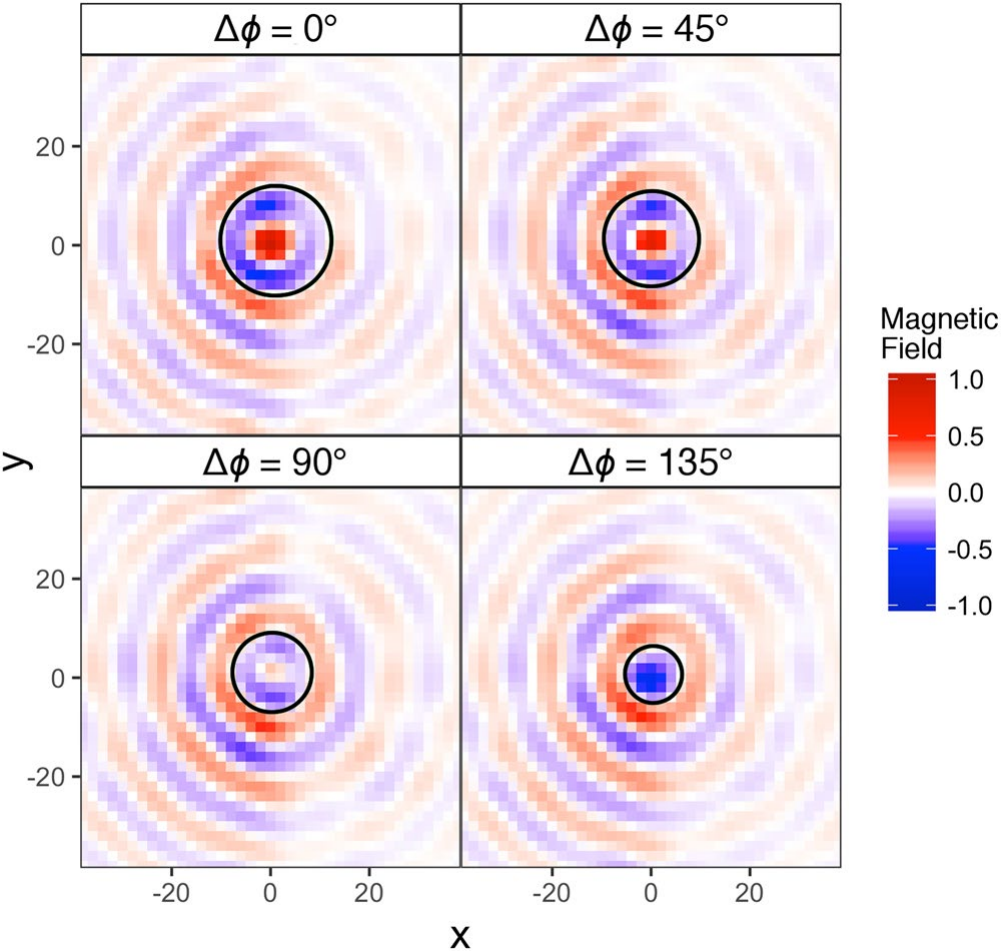
Measured instantaneous magnetic field (H_z_, arbitrary scale) at a height of 0.5 mm above the metasurface. The surface mode is excited at the centre of the image. The complex field is advanced in phase from 0 to 135° and the phase fronts (e.g. the null in electric field marked by the black circle) propagate towards the source (negative phase velocity) whilst, for our measurement system, power must flow away from the source (positive group velocity).

In order to observe the magnetic field distribution associated with the backward wave alone, the recorded field data needs to be filtered in wavevector since a positive index mode exists at the same frequency. An inverse Fourier transform is performed on the isofrequency contours after a windowing function is applied. This window reduces the Fourier amplitude within a given radius in *k*-space to zero, effectively removing the forward wave. The resulting real space magnetic field map is shown in Fig. 5. To observe the direction of phase front propagation the complex field is advanced in phase by some step, Δ*ϕ* such that the field after the *n*th step is given by

1 $${{\rm{H}}}_{{\rm{n}}}={{\rm{H}}}_{0}\exp (i\varphi +{\rm{\Delta }}\varphi ).$$

Fig. 5 shows the real part of this complex field in the vicinity of the source position for values of Δ*ϕ* between 0° and 135°. With advancing phase, the phase fronts move towards the source indicating a backward wave, whilst the circular wavefronts demonstrate the isotropy.

## Conclusion

In this paper, the near-field excitation and field-mapping of a bound surface wave on a spiral metasurface has demonstrated that, across a narrow band of frequencies, both a forward and backward wave are supported. The backward wave represents a 2D analogy of waves within a negative index medium, with group and phase velocities in opposite directions. The array of spirals realises an approximation to an array of out-of-plane magnetic dipoles^25^, supporting a transverse magnetic mode that disperses with a negative gradient.

In recent years the manipulation of surface waves supported by metasurfaces has been a topic of extensive research resulting in graded index lenses and cloaking devices at microwave frequencies. The work presented here expands on this topic and adds isotropic negative mode index to the list of available properties when designing metasurface devices. In particular this research could enable negative refraction of surface waves and subsequently the development of a perfect lens for surface waves.

### Data Availability

All data created during this research are openly available from the University of Exeter’s institutional repository at https://ore.exeter.ac.uk.
